# Quality of life among Tunisian women across different menopausal stages

**DOI:** 10.3389/frph.2025.1687160

**Published:** 2025-11-19

**Authors:** Hela Snani, Haifa Snani, Fairouz Azaiez

**Affiliations:** 1 Higher Institute of Sport and Physical Education of Ksar-Said, Universite de La Manouba, Tunis, Tunisia; 2 Department of Education, Higher Institute of Sport, and Physical Education of Gafsa, University of Gafsa, Gafsa, Tunisia

**Keywords:** premenopause, perimenopause, postmenopause, quality of life, MENQOL, physical activity, BMI

## Abstract

**Introduction:**

Menopause represents an important transition in women's lives, often accompanied by physiological, psychological, and lifestyle changes that can affect overall quality of life (QoL). Understanding how individual characteristics, body composition, and daily habits influence QoL during this stage is essential. This study aimed to assess the prevalence of menopausal symptoms and to examine how anthropometric measurements, sociodemographic factors, and lifestyle variables—particularly physical activity levels—independently influence the QoL of Tunisian women during mid-life.

**Methods:**

A cross-sectional study was conducted among 375 women recruited from different regions of Tunisia and classified according to the World Health Organization's definitions of menopausal stages. Data were collected using the Menopause-Specific Quality of Life Questionnaire (MENQOL) and the Physical Activity Self-Assessment Questionnaire (J. Ricci ' L. Gagnon). Anthropometric measurements were performed by the research team. The Mann–Whitney and Kruskal–Wallis tests were applied to compare MENQOL scores across menopausal stages.

**Results:**

Among premenopausal women, the most reported symptom was feeling tired or worn out (76%), with the psychosocial domain showing the greatest QoL impact (score: 2.05), followed by physical (2.03), sexual (1.64), and vasomotor (1.36) domains. In perimenopausal women, fatigue was reported by 81%, with the physical domain most affected (2.03), followed by psychosocial (1.87), vasomotor (1.77), and sexual (1.28) domains. In postmenopausal women, flatulence or gas-related colic was the most frequent symptom (86%), and the psychosocial domain had the highest impact (2.36), followed by physical, vasomotor, and sexual domains. Overall, 68% of participants had a moderate level of physical activity. Higher physical activity was associated with better QoL in the total sample but remained significant only among premenopausal women. Overweight (42%) and obesity (28%) were common, and higher BMI was consistently linked to poorer QoL, particularly during premenopause.

**Discussion/Conclusion:**

Physical and psychosocial symptoms were the most prevalent across menopausal stages. While higher physical activity and lower BMI were associated with better QoL in premenopausal women, these associations weakened during menopause, suggesting that other factors, such as income and BMI, may play a stronger role in postmenopausal QoL. Promoting physical activity and healthy weight management could therefore improve QoL outcomes for women throughout the menopausal transition.

## 1 Introduction

Traditionally, the human body has been viewed through a medical lens. However, in recent decades, it has increasingly drawn the attention of specialists such as psychologists, sociologists, and anthropologists. This shift occurred as scientists came to understand that human beings cannot be studied in isolation from the environment in which they live. Psychological, cultural, and social interactions play a vital role not only in shaping a person's development but also in influencing their physical and mental health—in other words, their overall well-being. The World Health Organization (WHO) defines health as a complete state of physical, mental, and social well-being, not merely the absence of disease (1). Health after the age of 50 years has become a new concern to women in developed countries (2). With the global rise in life expectancy, many women now live more than two decades after menopause, spending over a quarter of their lives in a state of estrogen deficiency (3). However, after the mid-forties, nearly all women, regardless of their cultural background or health status, begin to experience physical, psychological, and emotional changes (4).

Menopause marks the time when a woman's menstrual periods have ceased for 12 consecutive months after the last period and is characterized by ovarian failure, which results to a decline in the production of the ovarian hormones estrogen and progesterone. During this phase women adapt to a new biological state. The deficiency of these hormones makes women susceptible to a range of common symptoms, including sleep disorders, mood swings, hot flashes, depression, urinary infections, vaginal infections, and an increased risk of osteoporosis and cardiovascular diseases. In addition to these physical symptoms, women are also inclined to experience several vasomotor, somatic, sexual, and psychological symptoms. The duration, severity, and impact of these symptoms can vary widely from person to person and among various populations which can impact their quality of life (QOL) (5). The World Health Organization (WHO) defines QoL as “an individual's perception of their position in life, in the context of the culture and value system in which they live, and in relation to their goals, expectations, standards, and concerns” (6). Menopause-related symptoms have a negative impact on the QoL of the late premenopause status women (7). Budakoglu et al. showed that the QoL in postmenopausal women is worse than that of premenopausal women (8).

To assess these changes and their impact, several tools have been developed, such as the MENQOL questionnaire. The Menopause-Specific Quality of Life Questionnaire (MENQOL) was first developed by Hilditch et al. in 1996 to capture the quality of life of midlife women over the past month. It has been translated and validated in multiple languages and applied in various epidemiological and clinical contexts. The self administered questionnaire explores four key domains: vasomotor, psychosocial, physical, and sexual. It evaluates both the presence and severity of a range of menopausal symptoms experienced by women (9). Several factors can significantly influence a woman's quality of life during menopause, including body mass index (BMI) and exercise. These factors not only affect the physical symptoms experienced but also play a crucial role in shaping the emotional and psychological well-being of women during this transition. Body mass index (BMI) is a widely used indicator of obesity status in clinical settings and population health research. According to the World Health Organization, BMI greater than 30 kg/m^2^ is considered obese. Sixteen articles were identified that specifically assessed and reported on the severity of menopausal symptoms in relation to measures of obesity including body mass index (BMI), waist circumference, and waist-to-hip ratio (10). Although weight gain during midlife is generally linked to aging rather than menopause itself (11, 12), gaining weight can negatively impact quality of life (12, 13). Moreover, physical activity (PA) has been shown to improve the QoL among menopausal women, likely due to its positive effects on neuroendocrine balance and the release of endogenous opioids, which help reduce vasomotor symptoms (14, 15).

Moreover, it can enhance psychosomatic well-being by enhancing self-esteem, improving sleep quality, and decreasing musculoskeletal pain and menopausal complaints (16, 17). Tunisia is a small country located on the Mediterranean Sea's southern coast in North Africa Despite being a part of the Arab world and having Arabic as its official language, Tunisia has a unique cultural identity that has been shaped by the Mediterranean and its diverse history. The country has made significant progress in women's rights and gender equality compared to many neighboring nations. Modern Tunisian society is greatly influenced by the active participation of Tunisian women in politics, the workforce and education. Despite the interest of researchers worldwide about the extent and type of symptoms experienced by women around menopause, only few studies have assessed this issue in our country. Ferrand et al. (18) (2013) provided a comparative study of Tunisian women and French women, showing that Tunisian women had reported lower quality of life, particularly in the dimension of life representing physical and psychological health. Delanoë et al. (2012) also presented a study of cross-cultural differences and found that Tunisian women presented more severe menopausal symptoms than French women (19).

Zaouali and Ben Slama (2023) reported on anxiety and depression among menopausal Tunisian women, addressing mental health issues at this transition (20). Abdelmoula et al. (2022) presented sexual dysfunction and satisfaction in post-menopausal women (21). The available literature on the QOL of menopausal women is primarily derived from Asian and industrialized Western populations. There is currently very little evidence derived from Arab countries and an extreme paucity in regard to data on the QOL of menopausal women in Tunisia. In fact, no studies exploring the effect of BMI and Exercise on QOL about Tunisian menopausal women. The proposed study will be the first. The aim of this study was to assess the menopausal related symptoms and their impact on the women's quality of life.

## 2 Materials and methods

### 2.1 Study design and sample

This cross-sectional research was conducted among Tunisian females, aged between 40 and 65 years old, recruited from various geographic districts (urban and rural) of Tunisia, on different sites such as medical clinics, work places, banks, schools and housewives living in households. The minimum calculated sample size was 375. Exclusion criteria included women with induced menopause, those who had undergone a simple hysterectomy, individuals currently receiving hormonal therapy, and those with pre-existing medical conditions such as diabetes mellitus, hypertension, cardiac disease, and thyroid disorders, as these factors could potentially confound the results. Pregnant or breastfeeding women and those suffering from mental illnesses, cognitive impairments or physical handicaps were not eligible for participation. Participants were categorized into three groups based on their menopausal status: premenopause stage (*n* = 259), menopausal transition (*n* = 93), and post menopause (*n* = 55).

### 2.2 Study instruments

In review the study employed a structured questionnaire administered through face-to-face to gather comprehensive data from each participant. The questionnaire was designed in three parts. The first section focused on collecting sociodemographic information and stage of menopause.The second part involved the MENQOL questionnaire. It consists of a total of 29 items, divided into four domains: vasomotor (items 1–3), psychosocial (items 4–10), physical (items 11–26) and sexual (items 27–29). Answers, provided in a Likert-scale format, were displayed as “no” or “yes”, with the latter spread from zero to six, respectively indicating the presence of the symptom and its degree, from being not bothersome to extremely bothersome. Calculations of each domain are computed separately and then summed up to reach the final score of MENQOL, as elucidated by the main developer of this tool (22) The third section included the Ricci & Gagnon self-assessment questionnaire, which focus on the Global assessment of activity and sedentary behavior with a Point system leading to inactive, active, very active categories. (BMI) was calculated as the actual weight, in kilograms, divided by height, in meters squared, relying on the anthropometric inputs (height, weight) measured respectively by a stadiometer and a digital scale, by the research team, the day of the recruitment. It was then categorized according to the WHO cut-off points: underweight if less than 18.5, normal if between 18.5 and 24.9, overweight if between 25 and 29.9 and obese from 30 and above (23).

### 2.3 Data collection procedure

The study was approved by the Ethics Committee on 21/03/2022 under the number No. 113 (Ref: CEFMS 113/2022) for our research project. Participants who met the inclusion criteria were informed in detail about the study and invited to participate voluntarily. Clear guidance on how to complete each section of the questionnaire was provided to all participants. They were also explicitly reassured that their privacy, anonymity, and the confidentiality of their responses would be strictly protected. Participants were informed that they were under no pressure to complete the questionnaire, had the right to withdraw from the study at any time without consequence, and were assured that their names would not be recorded or linked to their responses. Verbal informed consent was obtained from each participant prior to their inclusion in the study.

### 2.4 Validity and reliability of the MenQol Questionnaire

**Figure 1:**
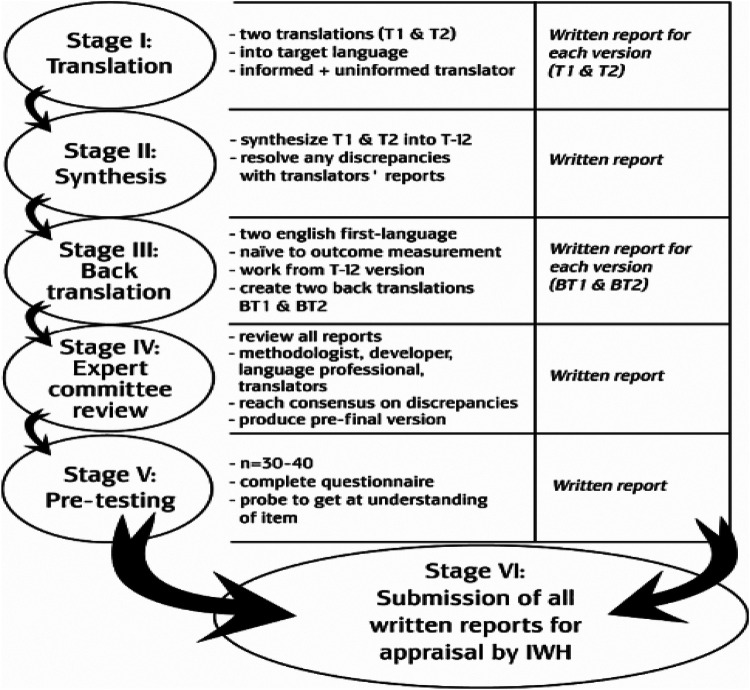
The different stages of cross-cultural adaptation recommended by the AAOS (American academy of orthopaedic surgeons) (Beaton et al. 2002).

The six-step cross-cultural adaptation process is illustrated in Figure 1. To ensure accurate translation of a questionnaire, it is crucial to have two teams of 2-3 translators each to translate it into the target language. These translators should be native speakers of the target language in two teams of 2–3 translators, each native speakers of the target language native speakers of the target language, fluent in its regional variations and idioms, and possess a strong understanding of the source language. One team should be familiar with the questionnaire's purpose and applications, while the other should be naive to its research goals and ideally have no background in the humanities to provide a more natural, target-population-oriented translation.

The resulting translated questionnaire then needs to be reviewed for accuracy and tested for clarity. Although the validity of the MENQOL questionnaire has been tested in Arab women (24). Its validity has not been previously tested in Tunisian women. Therefore, we tested its validity and reliability before starting the study using the test-retest method. “The MENQOL questionnaire was administered to the same population (*n* = 70) at the beginning of the study and then re-administered after 2 weeks.” The psychometric evaluation of the Arabic MENQOL in Tunisian women demonstrated satisfactory preliminary results, supporting the original four-factor structure (Vasomotor, Psychosocial, Physical, and Sexual) through both exploratory and confirmatory factor analyses.

The overall scale showed excellent internal consistency (McDonald's omega = 0.945) and good temporal stability (*r* = 0.879). Confirmatory factor analysis indicated a good fit to the data, and convergent validity was satisfactory, the Cronbach's alpha for the sexual domain was notably low, suggesting a potential area for improvement. These findings provide initial support for the use of the Arabic MENQOL in Tunisian menopausal women.

### 2.5 Statistical analysis

SPSS V.25 was used to analyze the collected data. Means and standard deviations were used to describe the continuous variables and percentages to describe categorical variables. The Kolmogorov–Smirnov test showed that data were not normally distributed; the Mann–Whiney U test and the Kruskal–Wallis test were used to compare MENQOL item scores. Multivariable linear regression analysis was used to assess predictors of the total MENQOL score of the study participants. Results were considered statistically significant at *p* < 0.05 (Table 1).

**Table 1:** Sociodemographic characteristics of the study participants.

Characteristic	*N*	% mean ± SD
Age (years)	–	47,4 ± 7,2
BMI		
Underweight	4	1,1
Normal weight	110	29
Over weight	158	42
Obesity	103	28
Marital status		
Single	35	9,3
Divorced	24	6,4
Maried	302	81
Widow	14	3,7
Education level		
Primary education	10	2,7
Secondary education	88	24
University education	277	74
Income		
Low	28	7,5
Meduim	258	69
High	89	24
Smoker		
No	305	81
Yes	70	19
Physical activity level		
Inactive	116	31
Active	256	68
Very active	3	0,8

## 3 Results

### 3.1 Effect of sociodemographic characteristics and quality of life domains

The study participants exhibited a notable proportion of overweight (42%) and obese (28%) individuals. The majority were married (81%) and well-educated, with 74% holding a university degree. Economically, most reported a medium income (69%). A significant majority were non-smokers (81%), and most engaged in some form of physical activity (68% active, 0.8% very active, 31% inactive) (Table 1).

Table 2 presents the premenopause group as the largest. Age, as expected, showed a clear increase across the stages. Body weight distribution was noteworthy, with a higher prevalence of overweight and obese individuals in the pre- and menopausal groups. Marriage was the most common marital status reported across all stages, though the proportion of single women was higher in the premenopause group, and widowhood became more apparent in later stages. A considerable proportion of the women had attained a university education. Income levels were largely concentrated in the medium category. The majority of participants were non-smokers and reported being physically active.).

**Table 2:** Characteristics of participants according to menopausal status (*n* = 375).

Characteristics	Menopausal status					
	Premenopause, *n*		Menopause, *n*		Post menopause, *n*	
Total (%)	259 (69,1)		93 (24,8)		23 (6,1)	
Age (years), mean ± SD	44 ± 3,941)		53,72 ± 6,2		60,26 ± 8,1	
BMI						
Underweight	2	0,5%	2	0,5%	0	0,0%
Normal weight	84	22,4%	20	5,3%	6	1,6%
Over weight	103	27,5%	44	11,7%	11	2,9%
Obesity	70	18,7%	27	7,2%	6	1,6%
Marital status						
Single	21	5,6%	14	3,7%	0	0
Divorced	22	5,9%	2	0,5%	0	0
Married	212	56,5%	69	18,4%	21	5,6%
Widow	4	1,1%	8	2,1%	2	0,5%
Education level						
Primary education	3	0,8%	4	1,1%	3	0,8%
Secondary education	48	12,8%	33	8,8%	7	1,9%
University education	208	55,5%	56	14,9%	13	3,5%
Income						
Low	17	4,5%	9	2,4%	2	0,5%
Medium	177	47,2%	65	17,3%	16	4,3%
High	65	17,3%	19	5,1%	5	1,3%
Smoker						
No	216	57,6%	71	18,9%	18	4,8%
Yes	43	11,5%	22	5,9%	5	1,3%
Physical activity level						
Inactive	89	23,7%	23	6,1%	4	1,1%
Active	168	44,8%	70	18,7%	18	4,8%
Very active	2	0,5%	0	0,0%	1	0,3%

### 3.2 Relationship between sociodemographic characteristics, physical activity, tobacco consumption and quality of life domains

Table 3 presents the associations between sociodemographic characteristics, physical activity, tobacco consumption and QoL domains of the study participants. In general, we found a statistically significant association between the total QoL score and BMI (*P* = 0,017*), Education Level (0,001*), Income (0,022*), Smoker (*p* = 0,017*), and physical activity Level (*p* = 0,011*), menopause status (0,000).

**Table 3:** The associations between sociodemographic characteristics physical activity, tobacco consumption and QoL domains.

Variable	Vasomotor domain	Psychosocial domain	Physical domain	Sexuel domain	Total
BMI					
Underweight	1,83 (1,67)	3,07 (1,54)	2,25 (1,67)	3,33 (2,83)	2,62 (1,67)
Normal weight	1,05 (1,49)	2,01 (1,46)	1,79 (1,26)	1,40 (1,53)	1,56 (1,07)
Over weight	1,71 (1,88)	2,31 (1,58)	2,14 (1,25)	1,72 (1,71)	1,79 (1,19)
Obesity	2,47 (2,13)	2,77 (1,61)	2,78 (1,40)	2,08 (2,01)	2,52 (1,40)
*p*-value^b^	0,005*	0,045*	0,163	0,178	0,017*
Marital status					
Single	2,04 (2.16)	2,61 (1,83)	2,26 (1,48)	1,49 (1,84)	2,10 (1,36)
Divorced	1,61 (1,63)	2,74 (1,75)	2,25 (1,41)	1,17 (1,61)	1,94 (1,28)
Married	1,67 (1,90)	2,30 (1,54)	2,20 (1,34)	1,86 (1,79)	2,00 (1,28)
Widow	2,43 (2,23)	2,35 (1,41)	2,43 (1,18)	0,90 (1,16)	2,03 (1,02)
*p*-value^b^	0,467	0,549	0,862	0,032*	0,939
Education level					
Primary education	1,40 (1,90)	1,91 (1,98)	2,19 (1,82)	1,73 (2,14)	1,81 (1,65)
Secondary education	2,39 (2,11)	2,77 (1,64)	2,48 (1,41)	2,30 (2,14)	2,48 (1,41)
University education	2,11 (1,82)	1,64 (1,53)	1,41 (1,31)	2,14 (1,61)	1,41 (1,18)
*p*-value^b^	0,002*	0,015*	0,119	0,037*	0,001*
Income					
Low	2,86 (2,31)	2,96 (1,84)	2,53 (1,68)	2,64 (2,20)	2,75 (1,59)
Medium	1,64 (1,88)	2,36 (1,57)	2,24 (1,36)	1,75 (1,73)	2,00 (1,28)
Good	1,63 (1,79)	2,16 (1,48)	2,04 (1,18)	1,45 (1,70)	1,82 (1,07)
*p*-value^b^	0,032*	0,101	0,351	0,019*	0,022*
Physical activity level					
Inactive	1,91 (1,87)	2,75 (1,55)	2,48 (1,42)	1,89 (1,83)	2,26 (1,30)
Active	1,66 (1,95)	2,18 (1,56)	2,11 (1,30)	1,70 (1,76)	1,91 (1,25)
Very active	1,11	1,81	0,79	0,22	0,98
*p*-value^b^	0,260	0,003*	0,009*	0,163	0,011*
Smoker					
No	1,58 (1,82)	2,28 (1,56)	2,16 (1,32)	1,67 (1,73)	1,92 (1,22)
Yes	1,82 (2,20)	1,56 (1,62)	1,32 (1,45)	1,73 (1,96)	1,22 (1,43)
*p*-value^a^	0,005*	0,045*	0,163	0,178	0,017*
Menopause status					
Premenopause stage	1,47 (1,79)	2,38 (1,61)	2,12 (1,32)	1,50 (1,72)	1,86 (1,25)
Menopause	2,66 (2,09)	2,47 (1,48)	2,58 (1,41)	2,29 (1,82)	2,50 (1,25)
post menopause	0,93 (1,30)	1,66 (1,48)	1,82 (1,22)	2,32 (1,73)	1,68 (1,12)
*p*-value^b^	0,000*	0,049*	0,008*	0,000*	0,000*

a *p*-values based on the Mann–Whitney U-test. Statistical significance at *P* < 0.05.

b *p*-values based on the Kruskal–Wallis H-test. Statistical significance at *P* < 0.05.

*Bold values indicate statistical significance (where the *p*-value is less than 0.05) or the highest number, percentage, or mean score in the descriptive columns.

Table 3 reveals significant associations between sociodemographic characteristics, physical activity, tobacco consumption and quality of life (QoL) domains. Specifically, total QoL was significantly associated with BMI, Education Level, Income, and Exercise Level.

Higher BMI generally correlated with worse Vasomotor and Psychosocial symptoms and lower total QoL. Lower Education Level was associated with poorer outcomes in the Vasomotor, Psychosocial, and Sexual domains, as well as lower total QoL.

Similarly, lower Income tended to be linked to worse Vasomotor and Sexual symptoms and lower total QoL. In contrast, higher Exercise Level was associated with better Psychosocial and Physical well-being and higher total QoL. Marital status showed a significant association with the Sexual domain. The different stages of menopause revealed significant differences in all domains (*p* < 0.05).

Women in the menopause stage reported the highest levels of impairment, particularly in the vasomotor (mean = 2.66, SD = 2.09) and physical (mean = 2.58, SD = 1.41) domains, compared to those in the premenopause and postmenopausal stages.

### 3.3 Quality of life domains among the study participants according to menopausal status

 Table 4 presents the frequency of Quality of Life (QoL) domains among the study participants. In the premenopause stage, the psychosocial domain imposed the greatest impact on quality of life (mean = 2.05), with Feeling anxious or nervous being predominant at 71%. Meanwhile, Night sweats emerged as the most prevalent vasomotor symptom at 47%. Concerning the physical domain, the most frequent complaint was Feeling tired or worn out, reported by 76% of women. In the sexual domain, Change in sexual desire was the most prevalent symptom, affecting 57% of participants. The mean total MENQOL score was 1.71, indicating a moderate level of reported quality of life impairment.

**Table 4:** Quality of life domains among the study participants according to menopausal status.

Quality of life domains	Premenopause stage *n* = 85				Menopause transition *n* = 266				Post menopause *n* = 55			
	*n*	%	Mean	(SD)	*N*	%	Mean	(SD)	*N*	%	Mean	(SD)
Vasomotor symptoms			**1,36**	1,658			**1,77**	1,973			**2,23**	2,114
Hot flashes or warmth	108	42%	1,39	1,908	47	51%	1,46		10	45%	2,86	2,232
Night sweats	122	**47%**	1,52	1,95	45	48%	1,86	2,253	14	64%	2,18	2,281
Excessive sweating	81	31%	1,14	1,935	47	51%	1,99	2,305	9	41%	1,73	2,354
Psychosocial			**2,05**	1,463			**1,87**	1,401			**2,59**	1,681
Dissatisfaction with personal life	88	34%	1,32	2,082	35	38%	1,28	1,93	11	50%	1,86	2,295
Feeling anxious or nervous	183	**71%**	2,58	1,991	66	**71%**	2,7	2,084	16	**73%**	3,14	2,232
Experiencing poor memory	168	65%	2,01	1,813	57	61%	1,62	1,713	17	**77%**	2,64	1,941
Accomplishing less than they used to	165	64%	2,11	1,859	61	66%	2,02	1,824	17	**77%**	3	2,07
Feeling depressed, down or blue	147	57%	1,97	2,052	59	63%	1,99	2,003	16	**73%**	2,23	2,137
Being impatient with other people	157	61%	2,02	1,973	48	52%	1,7	1,96	15	68%	2,45	2,283
Feelings of wanting to be alone	167	64%	2,26	2,178	53	57%	2,02	2,216	17	**77%**	2,82	2,152
Physical			**2,03**	1,349			**2,03**	1,355			**2,36**	1,677
Flatulence (gas) or colic caused by gas	165	64%	2,09	1,951	57	61%	2,27	2,101	19	**86%**	3	2
aches in the muscles and joints	174	67%	2,41	2,077	65	70%	2,58	2,097	13	59%	2,18	2,26
Feeling tired or worn out	196	**76%**	2,85	1,986	75	**81%**	2,89	2,035	18	**82%**	3,36	2,128
Insomnia and difficulty sleeping	142	55%	1,88	2,057	56	60%	2,24	2,144	53	41%	2,41	2,239
aches in the back, neck, or head	189	**73%**	2,67	2,045	67	**72%**	2,62	2,111	16	**73%**	2,73	1,98
Decrease in physical strength	171	66%	2,3	2	62	67%	2,03	1,897	16	**73%**	2,77	2,245
Decrease in endurance or ability to perform usual activities	147	57%	1,97	1,987	56	60%	1,91	1,965	16	**73%**	2,64	1,989
Loss of physical energy	170	66%	2,13	1,92	56	60%	1,88	1,983	15	68%	2,68	2,212
Dryness of the skin	136	53%	1,7	1,939	49	53%	1,78	1,966	16	**73%**	2,14	2,1
Weight gain	148	57%	1,94	2,012	54	58%	1,91	1,965	16	**73%**	2,64	1,989
Increased rate of hair loss	149	58%	2,05	2,09	55	59%	1,92	2,06	17	**77%**	2,86	2,336
Changes in appearance, texture or tone of skin	129	50%	1,65	1,93	47	51%	1,54	1,821	13	59%	1,73	1,882
Feeling bloated	156	60%	2,14	2,11	50	54%	1,87	1,996	18	**82%**	2,5	2,064
Low backache	177	68%	2,57	2,118	56	60%	2,18	2,206	15	68%	2,41	2,108
Frequent urination	102	39%	1,42	1,95	40	43%	1,18	1,594	14	64%	1,95	2,149
Involuntary urination when laughing or coughing	63	24%	0,61	1,323	26	28%	0,59	1,236	15	68%	2,05	2,236
Sexual			**1,64**	1,626			**1,28**	1,631			**2,05**	2,42
Change in sexual desire	148	**57%**	1,97	2,069	42	45%	1,54	2,003	11	50%	1,95	2,535
Dryness in the vagina during sexual intercourse	105	41%	1,3	1,878	27	29%	0,9	1,694	10	45%	2,14	2,606
Avoiding intimacy	121	47%	1,6	2,054	35	38%	1,39	2,157	10	45%	1,95	2,609
MENQOL			1,71	0,999			1,54	1,027			2,27	1,453

a *p*-values based on the Mann–Whitney U-test. Statistical significance at *p* < 0.05.

b *p*-values based on the Kruskal–Wallis H-test. Statistical significance at *p* < 0.05.

Bold values indicate statistical significance (where the *p*-value is less than 0.05) or the highest number, percentage, or mean score in the descriptive columns.

During the menopause stage, the physical domain imposed the greatest impact on quality of life (mean = 2.03), with Feeling tired or worn out being the prevailing complaint at 81%. In the psychosocial domain (mean = 1.87), Feeling anxious or nervous remained prominent at 71%, while Hot flashes or warmth and Excessive sweating, both at 51%, were the most commonly reported vasomotor symptoms. In the sexual domain, Change in sexual desire persisted as the most prevalent symptom, affecting 45% of participants. The mean total MENQOL score decreased to 1.54, suggesting a lower overall burden of reported quality of life impairment in this stage. In the postmenopausal stage, the psychosocial domain imposed the greatest impact on quality of life (mean = 2.59), with Experiencing poor memory, Accomplishing less than they used to, and Feelings of wanting to be alone each reported by **77%** of women. For physical symptoms (mean = 2.36), Flatulence (gas) or colic caused by gas (86%) and Feeling tired or worn out (82%) were most commonly reported. In the vasomotor domain, Night sweats emerged as the most prevalent symptom, reported by 64% of participants. In the sexual domain, Change in sexual desire remained the most prevalent symptom at 50%. The mean total MENQOL score increased to 2.27, indicating a greater burden of reported quality of life impairment in this stage.

### 3.4 Role of income, BMI, and physical activity level in predicting quality of life in menopausal women

Table 5 presents the Multivariable linear regression analysis used to assess predictors of the total MENQOL score. We found that the best-fitting predictors for the MENQOL score of our participants were income (*p* = 0.012), physical activity (*p* = 0.014), and BMI (*p* = 0.000). Specifically, participants with higher income had a MENQOL score that was, on average, 0.296 points lower than those with lower income (95% confidence interval −0.527 to −0.065). Similarly, more physically active menopausal women had a MENQOL score that was, on average, 0.327 points lower than less active women (95% confidence interval −0.588 to −0.066). Conversely, for every 1 kg/m^2^ increase in BMI, there was an average increase in the MENQOL score of 0.369 points (95% confidence interval 0.209–0.529). The R-squared coefficient of determination (*R*^2^ = 0.127) indicates that approximately 12.7% of the variability in the total MENQOL score is explained by this mode.

**Table 5:** Multivariable linear regression analysis of determinants of the total menopause-specific quality of life score.

Variable	B	Standard error	*T* statistic	*p*-value 95%	Confidence interval lower 95%	Confidence interval upper 95%
Constant	1,303	0,605	2,153	0,032	0,113	2,494
Age	0,007	0,009	0,808	0,42	−0,01	0,024
Income	−0,296	0,117	−2,516	0,012*	−0,527	−0,065
Physical activity level	−0,327	0,133	−2,461	0,014*	−0,588	−0,066
BMI	0,369	0,081	4,543	0,000*	0,209	0,529

* Bold values indicate statistical significance at *p* < 0.05; R^2^ = 0.127 or the highest number, percentage, or mean score in the descriptive columns..

Table 6 presents the Multivariable Linear Regression Analysis examining determinants influencing Menopause-Specific Quality of Life (MENQOL) scores across the different stages of menopause. In the premenopause stage, physical activity level (higher level associated with better QoL) and BMI (higher BMI associated with worse QoL) were significant predictors of total MENQOL. Interestingly, these significant associations were not observed in the perimenopausal stage and in the post-menopausal stage, while not reaching statistical significance at *p* < 0.05, trends indicated a potential association between higher income and better QoL, and between higher BMI and worse QoL.

**Table 6:** Multivariable linear regression analysis of determinants across the three stages of menopause-specific quality of life scores.

Determinants	Pre-menopause			Menopause			Post-menopause		
	*B*	*t*	*p*-value	*B*	*t*	*p*-Value	*B*	*T*	*p*-value
Constant	1,527	1,570	0,118	3,882	2,831	0,006*	2,891	1,168	0,259
Age	−0,006	−0,336	0,737	−0,030	−1,431	0,156	−0,032	−1,032	0,317
Income	−0,233	−1,650	0,100	−0,170	−0,713	0,478	−0,825	−1,946	0,068
Physical activity level	−0,338	−2,230	0,027*	−0,379	−1,275	0,206	0,128	0,226	0,824
BMI	0,388	4,050	0,000*	0,312	1,836	0,070	0,610	1,865	0,080
*R* ^2^	0,124			0,100			0,361		

* Bold values indicate statistical significance (where the *p*-value is less than 0.05) or the highest number, percentage, or mean score in the descriptive columns.

## 4 Discussion

The aim of this cross-sectional study was to assess the quality of life (QoL) of Tunisian women using the MENQOL questionnaire and to examine its association with physical activity level, anthropometric measures, sociodemographic characteristics, and lifestyle factors.

Previous studies have reported that the typical age range for menopause is between 45 and 55 years (3, 25). In our sample, the mean age at menopause was 53.72 years, which is higher than the 45-year median reported in the Réjiche study (20). This difference may reflect variations in population characteristics. Our sample likely included more urban, educated, and higher-income women—factors commonly associated with a later onset of menopause. This average age was also higher than that reported in the United States (51 years), Russia (50 years), Greece (49 years), Turkey and Egypt (47 years), and India (46 years) (26). The later onset observed in our population may also be partly explained by the participants' weight profile, as 42% were overweight and 28% were obese. These findings are consistent with a meta-analysis of 11 prospective studies showing that overweight and obese women had a 52% and 54% higher risk, respectively, of experiencing late menopause compared to women with a normal BMI (27).

Menopausal status was determined based on self-reported menstrual history and classified as premenopausal (regular cycles), perimenopausal (irregular cycles but not yet 12 consecutive months without menstruation), or postmenopausal (no menstruation for at least 12 consecutive months). Women with induced menopause, due to surgical or medical treatments, were excluded from the analysis.

Among the sociodemographic factors, marital status emerged as a significant predictor, particularly influencing the sexual domain. This finding is consistent with Ferrand et al. (2013) (18), who reported lower overall QoL in Tunisian women without partners. However, other studies have reported mixed results. Sievert et al. (2007) (28) found fewer vasomotor symptoms among partnered women in the US and Spain, while Loe et al. (2005) (29) found no significant relationship in Singapore. In Tunisia, as in many societies, sexuality and emotional intimacy are closely linked to marital relationships. The absence of a partner may lead to reduced sexual activity, lower desire, and feelings of loneliness, all of which negatively impact sexual well-being. In contrast, emotional support from a partner can enhance self-esteem, reduce stress, and improve overall QoL. Cultural differences regarding sexual openness and relationship dynamics may explain the variability between our findings and those from other settings.

BMI had a significant effect on vasomotor, psychosocial, and total QoL domains. This result is consistent with existing evidence (30, 31) indicating that higher BMI is associated with more severe menopausal symptoms and lower overall QoL. Maintaining a healthy weight may therefore be an important strategy for improving menopausal well-being.

Education level was another strong determinant. Most participants (74%) held a university degree, and higher education was associated with better vasomotor, sexual, and total QoL scores. These findings support earlier studies (19, 32, 33) showing that education promotes health awareness and better coping strategies during menopause. Interestingly, women with secondary education reported more severe symptoms than those with only primary education, possibly due to occupational stress or lifestyle factors not captured in our analysis.

Economic status, represented by income level, also influenced QoL. Women with lower income experienced more severe vasomotor and sexual symptoms, while higher income was associated with better outcomes. Financial resources may facilitate healthier lifestyles and greater access to healthcare, contributing to improved QoL. This observation aligns with Fahami's (2010) (34), findings linking socioeconomic status with more favorable menopausal experiences.

Physical activity emerged as a protective factor. Using the Ricci and Gagnon scale, we found that higher activity levels were associated with better psychosocial, physical, and total QoL scores. These results agree with prior studies (35, 36). demonstrating that regular physical activity alleviates menopausal symptoms and enhances well-being. To our knowledge, this is among the first North African studies to use a validated physical activity assessment in middle-aged women.

Smoking, on the other hand, was negatively associated with vasomotor, psychosocial, and overall QoL. This finding is consistent with previous research (37, 38) showing that smokers experience more frequent and intense hot flashes and night sweats. The relatively low smoking rate in our study (20%) may reflect social norms in Arab societies, where smoking among women remains culturally discouraged.

Menopausal status significantly influenced QoL in all domains. Women in menopause experienced the greatest symptom burden, particularly in vasomotor and physical domains, while sexual symptoms persisted into postmenopause. This trend has also been observed in Kazakhstan and Iran (39, 40). Overall, menopause-related symptoms can substantially impair women's personal, social, and emotional functioning (41). In our sample, physical and psychosocial domains were the most affected, consistent with studies from Qatar and the Gulf region (42). In contrast, research from Saudi Arabia, Jordan, and Iran (43, 44) has found sexual symptoms to be more prominent, reflecting cultural variations in health perception and reporting.

Fatigue was reported by 76% of premenopausal, 81% of perimenopausal, and 82% of postmenopausal women, consistent with hormonal changes that influence sleep and energy regulation (45, 46). Gastrointestinal symptoms, particularly bloating and flatulence, were also common, especially among postmenopausal women, possibly due to hormonal fluctuations affecting gut motility and microbiota (47, 48).

The psychosocial domain had the greatest impact on QoL, with poor memory, reduced productivity, and feelings of isolation reported by 77% of participants. These findings are consistent with prior studies describing cognitive and emotional difficulties, often referred to as “menopausal brain fog,” as well as increased social withdrawal (49, 50). Changes in sexual desire were the most frequently reported symptoms in the sexual domain (57%, 45%, and 50% across stages), likely reflecting both biological changes and the cultural reluctance to discuss sexual issues openly in Arab societies.

Vasomotor symptoms such as hot ashes and night sweats were relatively less frequent overall but increased in severity postmenopause. Sexual symptoms, including reduced desire and vaginal dryness, also worsened after menopause. These findings confirm that symptoms intensify as the menopausal transition progresses, particularly in the physical and psychosocial domains (53–66).

Variations in symptom frequency and severity can be attributed to differences in sociocultural context, ethnicity, genetics, and environmental exposures, which shape how women experience and manage menopause. Support from partners and families may also play an important role in coping and overall QoL.

In the multivariable regression analysis, lower income, higher BMI, and lower physical activity were independently associated with poorer QoL. While income remained a significant predictor overall, this association was less pronounced within individual menopausal stages, possibly due to smaller subgroup sizes and limited statistical power (51, 52).

Although our study included both urban and rural women, we did not conduct a separate statistical analysis comparing these groups. This decision was based on logistical constraints and unequal subgroup sizes, as most participants were recruited from urban health centers. Nevertheless, rural and urban environments may differ in several factors influencing menopausal experience, particularly diet and lifestyle. Rural women often consume more natural and plant-based foods, including legumes, grains, and vegetables rich in phytoestrogens and flavonoids—compounds with estrogen-like properties that may help relieve vasomotor and psychological symptoms. Conversely, urban women may have greater exposure to processed foods and more sedentary habits, potentially leading to higher BMI and more severe menopausal symptoms. The absence of detailed dietary data in our study is therefore a limitation, as nutrition may play a key role in symptom variability and overall QoL. Future studies should incorporate dietary assessments to better understand how environmental and nutritional factors influence menopausal health.

## 5 Strengths and limitations of the study

One of the main strengths of this study is that it is among the first to examine the quality of life of Tunisian women at various stages of menopause, while also assessing the role of physical activity using validated and culturally adapted tools. The MENQOL and Ricci & Gagnon questionnaires offered valuable insights into the physical, psychosocial, vasomotor, and sexual dimensions of menopausal experience. The inclusion of a broad range of sociodemographic, anthropometric, and lifestyle variables allowed for a more comprehensive understanding of the factors shaping women's well-being during this transitional period.

Another strength lies in the careful selection of participants. By excluding women with chronic conditions such as diabetes, hypertension, cardiovascular disease, or thyroid disorders, the study minimized potential confounding factors, ensuring that the observed associations were more likely to reflect the impact of lifestyle and sociodemographic variables.

However, the study also has some limitations. First, the relatively small sample sizes for the perimenopausal and postmenopausal groups may have limited our ability to detect significant differences in subgroup analyses. Second, because recruitment was primarily conducted in urban health centers, the sample included mostly urban women, limiting our ability to compare their experiences with those of women in rural settings who may have different lifestyles and health profiles.

Another important limitation is the absence of dietary data. Without information on food intake, we could not assess how nutrition—especially the consumption of phytoestrogens and flavonoids found in plant-based foods—may influence menopausal symptoms and body composition. This is particularly relevant in Tunisia, where rural diets tend to include more legumes, grains, and vegetables, which may help alleviate vasomotor and psychological symptoms, while urban diets are often higher in processed foods and linked to sedentary habits. Including dietary assessments in future research would help clarify these associations.

Lastly, as with any self-reported data, there is a risk of recall bias and social desirability bias, particularly on sensitive issues such as sexual health. Although confidentiality was emphasized, underreporting is possible. Future studies would benefit from integrating more objective or mixed-method approaches—such as dietary tracking or biological markers—to provide a fuller picture of the complex interactions between environment, lifestyle, and menopausal quality of life.

## 6 Conclusion

This study shows that menopause is not only a biological change but also a personal and social experience that strongly influences women's quality of life. Women who remain physically active, maintain a healthy weight, and benefit from emotional and social support experience fewer symptoms and better well-being.

Greater attention should be given to differences between urban and rural lifestyles, as well as to the influence of nutrition and culture on menopausal health. Health professionals should promote awareness and supportive programs that help women embrace this stage of life with confidence, balance, and dignity.

## Data Availability

The datasets presented in this article are not readily available because the dataset used in this study contains sensitive participant information and is subject to ethical and privacy restrictions. Access to the full dataset is limited and can only be provided upon reasonable request to the corresponding author, following approval from the relevant institutional review board and adherence to data protection regulations. Aggregated or anonymized data may be shared to support reproducibility of the results. Requests to access the datasets should be directed to HS haifa.sn.m1@gmail.com.
